# Effect of postoperative physical activity on the bottom-up attention of older patients who underwent lower extremity orthopedic surgery

**DOI:** 10.1097/MD.0000000000043823

**Published:** 2025-08-29

**Authors:** Kazuya Akiyama, Shinta Takeuchi, Kenichiro Takeshima, Yusuke Nishida

**Affiliations:** a Department of Rehabilitation Medicine, International University of Health and Welfare Narita Hospital, Narita, Japan; b Department of Physical Therapy, Okinawa College of Integrative Medicine, Urasoe, Japan; c Department of Physical Therapy, School of Health Sciences at Narita, International University of Health and Welfare, Narita, Japan; d Department of Orthopaedic Surgery, School of Medicine, International University of Health and Welfare (IUHW), Narita, Japan; e Department of Orthopaedic Surgery, International University of Health and Welfare (IUHW) Narita Hospital, Narita, Japan.

**Keywords:** bottom-up attention, physical activity, postoperative cognitive dysfunction

## Abstract

Increased postoperative physical activity is thought to be useful in preventing postoperative cognitive dysfunction (POCD) in elderly patients after lower extremity orthopedic surgery. This study aims to clarify the effect of postoperative physical activity on the course of bottom-up attention in elderly postoperative lower extremity orthopedic surgery patients. The study enrolled 22 patients aged ≥65 years who were admitted to the Hospital for total knee or total hip replacement surgery. The study design was a single-center longitudinal study. The subjects’ Posner task reaction time was measured preoperatively, postoperatively on day 7, and postoperatively on day 14. The amount of physical activity from postoperative on day 1 to postoperative day 14 was also measured using an accelerometer. Based on the measured physical activity, the subjects were divided into a high physical activity group (n = 11) and a low-physical activity group (n = 11) for statistical analysis. The results of 2-way analysis of variance revealed a significant interaction for reaction times to the Posner task in the invalid trials (*F*_2,20_ = 4.77, *P* = .02). Subsequent within-group 1-way analysis of variance indicated a substantial change in invalid trial reaction times in the high physical activity group (*F*_2,20_ = 6.85, *P* = .005). Multiple comparisons revealed significant improvements from preoperative measurements to postoperative day 14 in the high physical activity group (*t*_10_ = 3.81, *P* = .003). Between-group comparisons at each time point demonstrated significantly longer invalid trial reaction times in the low-physical activity group compared to the high physical activity group on postoperative day 14 (*t*_20_ = 2.92, *P* = .01). No significant differences were found in the Mini Mental State Examination at all-time points. In older patients who underwent postoperative lower extremity orthopedic surgery, those with high postoperative physical activity levels were less likely to have a decrease in bottom-up attention compared with those with low postoperative physical activity levels. Increased physical activity may be a beneficial physical therapy intervention in the prevention and improvement of postoperative cognitive dysfunction.

## 1. Introduction

Postoperative cognitive dysfunction (POCD) is a cognitive decline that begins a few days after surgery, and its clinical manifestations include cognitive dysfunctions such as impairments in memory, information processing, attention, and executive function.^[1]^ The prevalence of POCD increases in older people, and the risk of POCD development in older patients ranges from 25% to 40%.^[2]^ The 1-year mortality rate of patients with POCD is approximately twice that of patients without POCD. Additionally, POCD negatively impacts the quality of life and worsens the prognosis of the underlying disease.^[3]^ As the global population is expected to age in the future, countermeasures against POCD will become increasingly important.

Preoperative risk factors for POCD include advanced age, previous cerebrovascular disease, and preoperative cognitive decline,^[4]^ and intraoperative factors include invasive surgery and anesthetic effects.^[5]^ Postoperative factors include postoperative infections and respiratory complications.^[6]^ In addition, measures to prevent POCD are thought to be effective against effects on the central nervous system (CNS) caused by increased oxidative stress and microglial activation.^[7]^ Specific interventions include shortening preoperative fasting and drinking time, adopting minimally invasive surgical methods, using short-acting anesthetics, and implementing early postoperative release from bed.^[7]^ Previous studies have reported that physical activity affects the normalization of autophagy by suppressing microglial activation,^[8]^ affects the expression of brain-derived neurotrophic factors, and protects neurons.^[9]^ Accordingly, promoting early bed release and increasing postoperative physical activity are considered effective countermeasures for POCD. However, whether increasing postoperative physical activity is effective in preventing POCD is unclear. One of the reasons is the lack of a clear evaluation method for POCD.^[10]^ In this study, we focused on postoperative changes in spatial attention, particularly bottom-up attention, to confirm the signs of POCD.

Spatial attention is classified into bottom-up and top-down attention.^[11]^ Bottom-up attention is an externally guided process in which information is automatically selected to be processed because of the highly salient features of the stimulus. Top-down attention is an internally guided process in which information is actively retrieved in the environment based on spontaneously selected factors. The functional connectivity of the ventral attentional network that controls spatial attention, particularly bottom-up attention, is reduced even in older people with mild cognitive decline that does not manifest as clinical symptoms, suggesting that changes in bottom-up attention may reflect changes in cognitive function as a precursor or early stage of cognitive impairment.^[12]^ Therefore, in this study, we focused on changes in spatial attention, particularly bottom-up attention, to evaluate mild cognitive decline as a sign of POCD.

Conversely, measures against POCD are particularly important for older postoperative patients with osteoarthritis of the lower limbs. Osteoarthritis is a bone and joint disease based on age-related degenerative degeneration, and it interferes with activities of daily living because of pain and decreased walking ability. Among osteoarthritis, hip and knee osteoarthritis have significant functional disability, and the number of patients is expected to increase with the aging of the population.^[13]^ Osteoarthritis therapy can be roughly divided into conservative and surgical therapies. Conservative therapy includes medication, exercise, and bracing. Surgical therapy includes total knee arthroplasty (TKA) for knee osteoarthritis and total hip arthroplasty (THA) for hip osteoarthritis.^[14]^ In patients who underwent hip arthroplasty, including THA, postoperative re-fractures and deaths because of cognitive dysfunction are often reported. The risk of these adverse outcomes is particularly high due to cognitive impairment.^[15]^ Cognitive impairment was also reported to affect postoperative walking ability and prolong hospital stay in patients who had undergone knee arthroplasty.^[16]^ Studies reporting the long-term course of cognitive function in patients who underwent orthopedic and oncologic surgery have reported long-term persistence of cognitive decline in patients after orthopedic surgery.^[17]^ However, previous studies have not examined the association with the amount of physical activity in the early postoperative period. Older patients who underwent postoperative orthopedic surgery may find it difficult to increase their postoperative physical activity level because of preoperative functional impairment and postoperative functional decline in the lower limbs. Therefore, it is necessary to clarify the effect of postoperative physical activity on cognitive decline, especially bottom-up attention, in older patients after lower limb orthopedic surgery, such as postoperative TKA and THA patients.

In this study, we hypothesized that in patients who had undergone TKA and THA, those with high postoperative physical activity levels would show a decline in bottom-up attention than those with low postoperative physical activity levels. We focused on the decline in bottom-up attention as a sign of POCD and aimed to clarify how the level of postoperative physical activity affects the course of bottom-up attention in older patients who underwent orthopedic surgery of the lower limbs.

## 2. Methods

### 2.1. Participants

The study enrolled patients aged ≥65 years who were admitted to the International University of Health and Welfare Narita Hospital for TKA or THA surgery between January 27, 2022, and September 14, 2022. In a previous study, brain activity during the Posner task was reported in right-handed participants.^[18]^ Therefore, this study was also limited to right-handed participants. The exclusion criteria were as follows: difficulty performing the measurements before surgery, presence of upper limb dysfunction, visual field or vision impairment, a history of CNS disease, MMSE score (MMSE) of ≤26 before surgery, and inability to provide consent.

Postoperative rehabilitation for both TKA and THA was performed according to the rehabilitation protocol of the International University of Health and Welfare, Narita Hospital. Regarding the protocol, physical and occupational therapies were prescribed for both TKA and THA. For physiotherapy and occupational therapy, the participants were instructed to start bed rest and full weight bearing on the day after surgery. For physical therapy, patients get off the bed and performed joint mobilization, muscle-strengthening, basic movement, and gait exercises from the day after surgery. For occupational therapy, the patient was weaned from bed and practiced ADLs from the day after surgery. Physical and occupational therapies were generally conducted 40 minutes a day, 5 days a week. The duration of inpatient rehabilitation was generally from 14 to 21 days after surgery when the patients were transferred or discharged from the hospital.

### 2.2. Study protocol

This single-center longitudinal study collected data from January 27, 2022 to September 14, 2022. The study period was from January 27, 2022 to September 28, 2022.

Participants’ basic information, intraoperative findings, blood data, medication status, and ambulation were extracted from the electronic medical records. The reaction time to the Posner task was measured as an index of spatial attention; the reaction times to the incongruent and congruent conditions of the Posner task were used as an index of bottom-up attention and top-down attention, respectively. The number of steps taken was measured using a physical activity meter as a measure of physical activity. Physical activity was measured from day 1 to day 14 postoperatively. MMSE was measured on the same day as the Posner task reaction time, preoperatively, postoperative day 7, and postoperative day 14.

### 2.3. Measurements

#### 2.3.1. Basic information

Age, gender, height, weight, body mass index, disease name, comorbidity, and disease severity were extracted. The Kellgren–Lawrence grade for knee osteoarthritis and the Japanese Orthopaedic Association hip function score for hip osteoarthritis were used to determine disease severity.

#### 2.3.2. Intraoperative observations

Side of surgery, surgical procedure, intraoperative sedation time, operative time, and intraoperative hemorrhage.

#### 2.3.3. Blood data

C-reactive protein, hemoglobin, and total protein were extracted preoperatively and 5 days postoperatively. Preoperative albumin was also extracted. Because albumin was not measured postoperatively, only preoperative data were extracted.

#### 2.3.4. Medication status

The presence or absence of CNS medications was extracted from the medical records. CNS drugs included hypnotic sedatives, anxiolytics, antiepileptic drugs, and neuropsychiatric drugs.

#### 2.3.5. Ambulation

The level of ambulatory independence upon admission was extracted from the medical records as preoperative ambulation. The postoperative ambulation was measured as the number of days from the time of surgery to the time of walking independently in the ward, and this information was extracted from the medical records.

#### 2.3.6. Posner task reaction time

The Posner task, developed by Posner, is a method of quantifying spatial attention.^[19]^ PsychoPy^[20]^ was used to create the Posner task. PsychoPy is an application for building a psychological experiment environment based on the Python language that can perform everything from stimulus creation and response time collection to data analysis.

**Figure 1.**
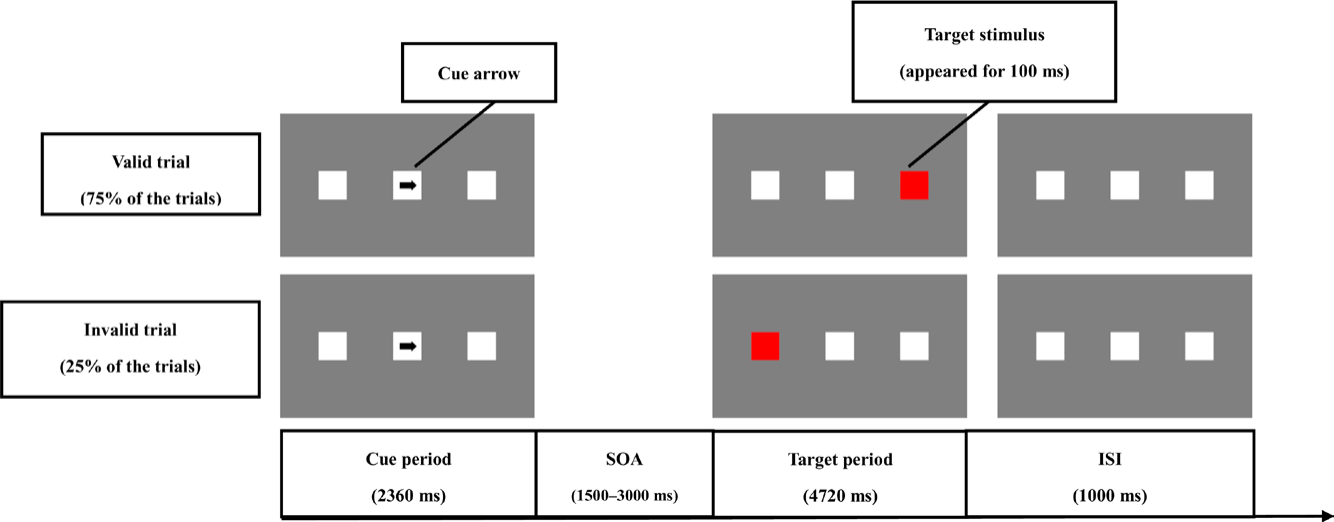
Setup of the Posner task. At the beginning of the trial, a central fixation point (square) and squares to the left and right of the fixation point were displayed. The squares on the left and right sides were displayed at a viewing angle of approximately 3.3°. After the start of the trial, a cued stimulus (arrow) pointing to the left or right square was displayed at the fixation point for 2360 ms. The left or right arrow was set to appear with equal probability. After the cue arrows were presented, target stimuli were randomly presented for 1500, 2000, 2500, and 3000 ms stimulus onset asynchrony (SOA), followed by a 100-ms period in which either the left or right square changed to red. In the valid trials (75% of trials), the square pointed by the cue arrow changed to red, and in the invalid trials (25% of trials), the square opposite to the square pointed to by the cue arrow changed to red. After the detection of the target stimulus or 4720 ms after the target stimulus was displayed, the next trial was set to start after an inter stimulus interval (ISI) of 1000 ms. ISI = inter stimulus interval, SOA = stimulus onset asynchrony.

Figure 1 shows the setup of the Posner task used in this study. The number of trials was set to 160 per session, and the trial types for the valid and invalid trials were set to appear randomly. The examiner instructed the participants to sit 60 cm away from the laptop display. The participant was also instructed to press the space key on the laptop as quickly as possible when the target stimulus was detected. The number of trials was 160 per session, and each session took approximately 10 minutes. The measurement site was a private room in the Speech and Hearing Room of Narita Hospital, International University of Health and Welfare, Narita, Japan. Before the measurement, services running on the laptop computer that were unnecessary for the experiment were stopped.

Previous studies have reported that cognitive function cannot be accurately assessed before 7 days postoperatively because of sedation and postoperative delirium.^[5]^ Therefore, the first postoperative Posner task reaction time should be measured on postoperative day 7. In addition, patients are transferred or discharged from Narita Hospital of International University of Health and Welfare after TKA and THA on or after postoperative day 14. We considered that the Posner task could be measured in all participants up to postoperative day 14. Therefore, the Posner task was measured preoperatively, postoperative day 7, and postoperative day 14.

#### 2.3.7. Physical activity

An accelerometer (Suzuken Corporation, Lifecorder, hereafter referred to as “Lifecorder”) was used to measure physical activity.^[21,22]^ The participants were told to wear the Lifecorder on their waist as much as possible during the measurement period. However, they were told to remove the Lifecorder when bathing or falling asleep. The measurement period was from postoperative day 1 to 14. The average number of steps taken per day from postoperative days 1 to 7, and from postoperative days 8 to 14 were used as indices of physical activity.

#### 2.3.8. Cognitive function

The MMSE is widely used internationally in clinical practice and research and consists of a total of 30 points: 29 points for tests of register, verbal memory, general attention, calculation, and language, all of which use verbal functions, and 1 point for graphic imitation. The MMSE has high intra- and inter-rater reliability and validity.^[23]^ In general, a cutoff score of ≤23 on the MMSE is considered suggestive of dementia,^[24]^ and a cutoff score of ≤26 on the MMSE is considered suggestive of mild cognitive impairment.^[25]^

### 2.4. Statistical analysis

The representative value of the reaction time for the Posner task was obtained by calculating the representative value of the reaction time for the congruent (60 trials on both sides) and incongruent (20 trials on both sides) conditions for each session obtained from the measurement and used for the statistical analysis. Outliers were removed from the mean, and reaction times exceeding 3 standard deviations from the mean were considered outliers.

A 1-way analysis of variance (ANOVA) was conducted to examine changes in Posner task reaction times across participants. The participants were then divided into groups to clarify the effect of postoperative physical activity on bottom-up attention. Regarding the grouping method, previous studies have reported that physical activity is beneficial to cognitive function^[8,26]^; however, how much physical activity is necessary to maintain cognitive function is unclear. Therefore, based on the hypothesis that participants with high postoperative physical activity would show less decline in bottom-up attention than participants with low postoperative physical activity, the top 11 participants with high physical activity from postoperative days 1 to 7 among all 22 participants were classified into the high physical activity group and the remaining 11 participants into the low-physical activity group. The lower 11 participants were categorized as the low-physical activity group.

After grouping, between-group comparisons were performed using *t*-tests with no correspondence for age, height, weight, body mass index, intraoperative blood loss, preoperative and postoperative C reactive protein, preoperative and postoperative hemoglobin, preoperative and postoperative total protein, and preoperative albumin. Between-group comparisons were performed using the Mann–Whitney *U* test for intraoperative sedation time, operative time, number of days to ward walking independence, MMSE (preoperative, postoperative day 7, and postoperative day 14), and physical activity (postoperative days 1–7 and postoperative days 8 to 14).

In addition, a 2-way ANOVA was conducted on the reaction time of the Posner task by activity × time of measurement. If an interaction was found, the level of the interaction was changed to 1 factor, and the ANOVA was performed again. The significance level for all statistical analyses was 5%. Statistical analyses were done using R software, version 1.4.1106.

### 2.5. Ethical considerations

All participants were informed of the purpose and methods of this study and details of the benefits and disadvantages to the participants, both orally and in writing, and their consent to participate in the study was obtained. This study was conducted after approval by the Ethics Committee of the International University of Health and Welfare (Approval no. 20-Io-182-2).

## 3. Results

### 3.1. Baseline information

**Figure 2.**
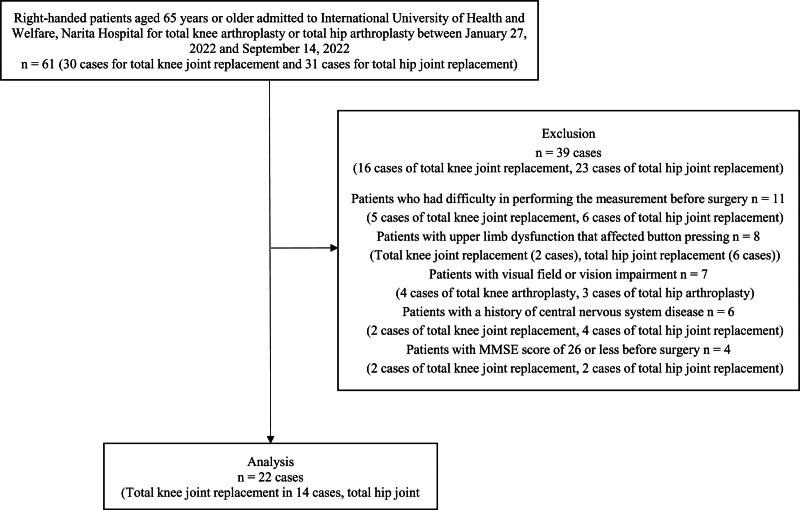
Flowchart of the study participants.

The analysis included 22 participants. A flowchart of the study participants is shown in Figure 2. No adverse events were observed in the study participants as a result of their participation in the study. In preoperative ambulation, all participants were walking independently, either independently or with the use of a walking aid. In postoperative ambulation, all participants can independently in walk in the wards using a walking aid. In addition, all participants had no clinical symptoms suggesting visuospatial impairment due to cognitive decline or decreased bottom-up attention in their ward life, nor were there any disorders of consciousness caused by postoperative delirium.

The results of intergroup comparisons of the baseline information, intraoperative findings, blood data, medication status, ambulation status, MMSE, and physical activity in each group are shown in Table 1. Significant differences were found only in physical activity on postoperative days 1 to 7 (*P* = .005) and 8 to 14 (*P* = .001). No significant differences were found in MMSE scores at all-time points.

**Table 1:** Basic information for each group.

		High physical activity group (n = 11)	Low physical activity group (n = 11)	Significant difference
Age (yr)		71.3 ± 5.3	74.2 ± 4.7	n.s.
Gender (%)	Male	27.3	9.1	
	Female	72.7	90.9	
Height (cm)		153.4 ± 7.5	154.4 ± 7.9	n.s.
Body weight (kg)		57.1 ± 7.4	53.3 ± 12.1	n.s.
BMI (kg/m^2^)		23.1 ± 3.1	23.3 ± 3.8	n.s.
Disease (%)	Knee osteoarthritis	63.6	63.6	
	Hip osteoarthritis	36.4	36.4	
Comorbidity (%)	Knee osteoarthritis	36.4	45.5	
	Hip osteoarthritis	0	18.2	
	Brittle bone disease	18.2	45.5	
	High blood pressure	36.4	27.3	
	Diabetes	36.4	18.2	
	TKA	9.1	18.0	
	THA	9.1	0	
	Lumbar spondylolisthesis	9.1	0	
	Pulmonary emphysema	9.1	0	
	Aortic stenosis	0	9.1	
	Chronic kidney disease	9.1	0	
KL grade (%)	KL 2	14.3	0	
	KL 3	14.3	71.4	
	KL 4	71.4	28.6	
Hip joint JOA scoreG (points)		53.0 ± 16.4	48.7 ± 6.1	
TKA procedure (%)	Trivector	57.1	71.4	
	Medial parapatellar	42.9	28.6	
THA procedure (%)	Antero lateral supine	100.0	100.0	
Intraoperative sedation time (min)		190.0 (170.5–199.5)	186.0 (160.0–199.5)	n.s.
Operation time (min)		131.0 (120.5–141.5)	111.0 (101.5–129.0)	n.s.
Intraoperative blood loss (mL)		152.5 ± 108.8	129.3 ± 110.1	n.s.
Preoperative CRP (mg/dL)		0.2 ± 0.1	0.1 ± 0.1	n.s.
Postoperative CRP (mg/dL)		10.2 ± 5.1	8.6 ± 3.1	n.s.
Preoperative Hb (mg/dL)		14.0 ± 1.1	13.0 ± 1.3	n.s.
Postoperative Hb (mg/dL)		10.4 ± 1.2	10.0 ± 1.3	n.s.
Preoperative TP (mg/dL)		7.3 ± 0.5	7.2 ± 0.4	n.s.
Postoperative TP (mg/dL)		5.6 ± 0.4	5.9 ± 0.3	n.s.
Preoperative ALB (mg/dL)		4.5 ± 0.3	4.3 ± 0.2	n.s.
Central nervous system drug users (%)		45.5	36.4	n.s.
Days until independent walking (d)		6.0 (5.0–6.5)	7.0 (6.0–8.5)	n.s.
Preoperative MMSE (points)		30.0 (29.0–30.0)	29.0 (29.0–30.0)	n.s.
Postoperative 7-d MMSE (points)		30.0 (29.0–30.0)	30.0 (29.0–30.0)	n.s.
Postoperative 14-d MMSE (points)		30.0 (29.5–30.0)	30.0 (29.0–30.0)	n.s.
Physical activity 1–7 d after operation (steps)		213.1 (168.7–315.8)	61.8 (39.4–69.1)	*
Physical activity 8–14 d after operation (steps)		1321.4 (506.4–2026.1)	222.9 (123.1–434.4)	*

Age, height, body weight, BMI, JOA score, intraoperative blood loss, CRP, TP, Hb, and ALB are shown as means ± standard deviations. gender, disease, comorbidity, KL classification, surgical procedure, and central nervous system drug use are shown as percentages.

Intraoperative sedation time, operation time, number of days to ambulatory independence, MMSE, and physical activity are shown as median (first and third quartiles).

ALB = albumin, BMI = body mass index, CRP = C-reactive protein, Hb = hemoglobin, JOA = Japanese Orthopedic Association, KL = Kellgren–Lawrence grade, MMSE = Mini Mental State Examination, n.s. = Not Significant, THA = total hip arthroplasty, TKA = total knee arthroplasty, TP = total protein.

* *P* < .05.

### 3.2. Results of the 2-way ANOVA of the reaction time to the Posner task

Table 2 shows the results of the 2-way ANOVA for the reaction time to the Posner task. An interaction was found between the reaction time in the invalid trials (*F*_2,20_ = 4.77, *P* = .02).

**Table 2:** Two-way ANOVA for each trial.

	Preoperative		Postoperative 7-d		Postoperative 14-d		Significant difference		
	High physical activity group	Low physical activity group	High physical activity group	Low physical activity group	High physical activity group	Low physical activity group	Group	Time	Group × time
Invalid trial (ms)	468.9 ± 51.7	499.2 ± 87.2	459.4 ± 67.4	529.1 ± 95.0	434.3 ± 48.4	522.4 ± 104.0	n.s.	n.s.	*
Valid trial (ms)	449.6 ± 51.4	469.3 ± 78.9	434.7 ± 58.6	496 ± 75.5	423.5 ± 50.5	484.4 ± 74.2	n.s.	n.s.	n.s.

n.s. = Not significant.

* *P* < .05.

### 3.3. Results of the 1-way ANOVA of the reaction time to the Posner task in each group

**Figure 3.**
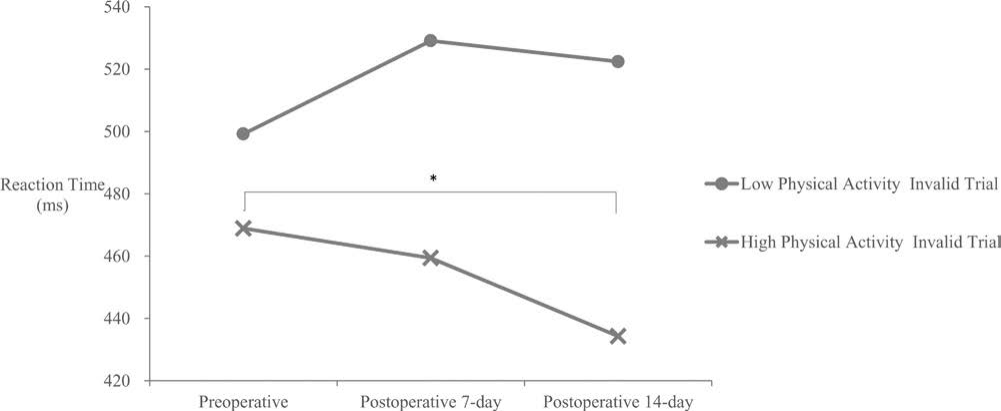
One-way ANOVA for the invalid trials in each group. The results of the 1-way ANOVA for the reaction time to the Posner task in the invalid trials for each group are shown. Significant differences were found in the invalid trials for the high physical activity group. Because significant differences were found in the invalid trials, a paired *t*-test using the Shaffer method was conducted as a multiple-comparison method. The results of multiple comparisons showed significant differences between preoperative and postoperative day 14 and between postoperative days 7 and 14. The low physical activity group showed no significant differences in any of the trials. * *P* < .05 (correspondence *t*-test using the Shaffer method). ANOVA = analysis of variance.

Since an interaction was observed for the invalid trials in the 2-way ANOVA, a 1-way ANOVA was conducted for each group. The results of the statistical analysis are shown in Figure 3. A significant difference was found in the invalid trials in the high physical activity group (*F*_2,20_ = 6.85, *P* = .005). Because a significant difference was found, a paired *t*-test using the Shaffer method was performed as a multiple-comparison method. The results of multiple comparisons showed significant differences between preoperative and postoperative day 14 (*t*_10_ = 3.81, *P* = .003).

### 3.4. Results of the comparison of the reaction times to the Posner task between groups at each time point

Because the 2-way ANOVA showed an interaction between the invalid trials, a *t*-test was conducted for each time point as a between-group comparison. The results of the statistical analysis are shown in Table 3. Significant differences were found in the invalid trials on postoperative day 14 (*t*_20_ = 2.92, *P* = .01).

**Table 3:** Results of the group comparisons of invalid trials at various time points.

	High physical activity group (n = 11)	Low physical activity group (n = 11)	Significant difference
Preoperative Invalid trial (ms)	468.9 ± 51.7	499.2 ± 87.2	n.s.
Postoperative 7-d Invalid trial (ms)	459.4 ± 67.4	529.1 ± 95.0	n.s.
Postoperative 14-d Invalid trial (ms)	434.3 ± 48.4	522.4 ± 104.0	*

n.s. = Not Significant.

* *P* < .05.

## 4. Discussion

Since POCD was reported to worsen the prognosis of the primary disease when it occurs as a symptom,^[3]^ a decline in cognitive function must be detected as a symptom before the appearance of major symptoms. In this study, we focused on bottom-up attention as a sign of POCD before it appears as a clinical symptom and examined how the amount of physical activity affects this sign.

In the MMSE results for all participants, the cutoff value for mild cognitive impairment was ≥26 points at all-time points. Therefore, the study participants did not show any preoperative cognitive decline based on the MMSE scores, and no noticeable clinical symptoms of POCD emerged after surgery. No significant difference in the preoperative reaction time to the Posner task was found between the 2 groups. Therefore, we believe in the lack of a clear difference in the preoperative bottom-up attention status of the participants in this study compared with healthy older participants.

The number of days to ambulatory independence in the ward was 6.0 days in the high physical activity group and 7.0 days in the low-physical activity group postoperatively. Previous studies have reported postoperative gait gains in 7.5 to 11.8 days^[27,28]^ in the TKA group. The number of days of walking independence in the THA group was 9.3 days.^[29]^ Based on these findings, the postoperative course of the study participants after TKA and THA was considered similar to that of the general results.

In the 2-way ANOVA and multiple-comparison for the reaction time to the Posner task, significant differences were observed in the reaction time for the invalid trial between preoperative and postoperative day 14. Additionally, in the between-group comparisons at each time point, the low-physical activity group had significantly higher reaction times for the invalid trial on postoperative day 14 compared to the high physical activity group. In a study in which multiple sessions of the Posner tasks were performed on unilateral spatial neglect patients, and progress in the reaction time was observed.^[30]^ The reaction time decreased as the number of sessions increased. In the discrepancy condition of the Posner task, the activity of the Temporo-parietal junction, a part of the ventral attentional network, was confirmed at the time of target stimulus detection.^[31]^ Therefore, the repetition of the Posner task during each measurement period possibly altered the synaptic transmission efficiency of the brain network involved in the implementation of the Posner task,^[32]^ resulting in a change in the reaction time. Surgical tissue damage was associated with the release of damage-associated molecular patterns,^[33]^ and damage-associated molecular patterns bind to Toll-like receptors and the receptor for advanced glycation end products^[34,35]^ and induce the production of inflammatory cytokines (interleukin [IL]-1, IL-6, IL-8, and tumor necrosis factor-α).^[36]^ Inflammatory cytokines induce vascular endothelial cells to produce Prostaglandin E2 and matrix metalloproteinases, resulting in weakened basement membranes and reduced expression of molecules necessary for tight junction formation, weakening the blood–brain barrier.^[37]^ Inflammatory cytokines pass through the weakened blood–brain barrier and activate microglia,^[38]^ and the activated microglia suppress autophagy, leading to amyloid-β accumulation, resulting in neuronal damage.^[39]^ In addition, autophagy failure leads to the accumulation of defective mitochondria, resulting in neuronal cell death caused by increased oxidative stress.^[40]^ In contrast to the above, physical activity affects the normalization of autophagy by suppressing microglial activation.^[8]^ Physical activity was reported to affect the expression of brain-derived neurotrophic factors and protect neurons.^[9]^ Therefore, in the low-physical activity group, neuronal damage caused by postoperative inflammation may cause functional impairment of the ventral attentional network, resulting in decreased bottom-up attention and higher reaction times to the invalid trial. Long-term cognitive decline has been reported following orthopedic surgery.^[17]^ In contrast, our study is novel in demonstrating that, during the early postoperative period, the amount of physical activity significantly influences bottom-up attention.

This study clarified how the amount of postoperative physical activity affects the course of bottom-up attention in older patients who had undergone lower extremity orthopedic surgery, focusing on the decline in bottom-up attention as a symptom of POCD. The results of the study revealed that the older patients who had undergone lower limb orthopedic surgery and had high postoperative physical activity levels showed a decline in bottom-up attention compared with those with low postoperative physical activity levels. Therefore, we were able to clarify the influence of postoperative physical activity on the course of bottom-up attention in older patients who had undergone lower limb orthopedic surgery.

In this study, we focused on bottom-up attention as a sign of POCD before it emerges as a clinical symptom. MMSE results between the high and low-physical activity groups showed no significant differences at all-time points. However, the reaction time to the Posner task showed no significant difference between the high and low-physical activity groups. Compared with the high physical activity group, the low-physical activity group showed significantly higher values for the invalid trial on postoperative day 14. Previous studies have reported reduced functional connectivity of the ventral attentional network controlling bottom-up attention in older participants with mild cognitive decline,^[12]^ and these results may capture the cognitive decline before the appearance of POCD symptoms. However, in this study, MMSE was only conducted up to 2 weeks postoperatively, and whether the reduction in bottom-up attention associated with the surgery affects cognitive decline over the long-term is unclear. Therefore, the decrease in bottom-up attention in the low-physical activity group can only be regarded as a possibility that POCD may be suspected.

Future studies must consider physical therapy intervention to prevent and improve POCD. The results of this study suggested that the amount of physical activity influenced the maintenance and improvement of bottom-up attention. Increasing physical activity may be a beneficial physiotherapy intervention in preventing and improving POCD, and its effectiveness must be verified.

This study focused on the decline in bottom-up attention as a sign of POCD and clarified the influence of postoperative physical activity on the course of bottom-up attention in older patients who had undergone lower limb orthopedic surgery. The results of this study will contribute to the development of evaluation and intervention methods for the prevention of POCD in older patients who had undergone lower limb orthopedic surgery.

## 5. Limitations of the study

This study has several limitations. First, the sample size was insufficient. Thus, increasing the sample size and conducting statistical analysis are necessary. Second, in the research method, eye-gaze measurement was not conducted during the Posner task, and whether the participants could maintain a fixed gaze point was unclear. In a previous study, gaze was maintained by verbally instructing older and unilateral spatial neglect patients to maintain gaze at a fixed point during the Posner task. In the present study, as in the previous study, the participants were verbally instructed to maintain gaze at a fixed point. However, the study participants may have impaired cognitive function during the postoperative period, and whether the verbal instructions were accurately conveyed was unclear. In the future, measuring eye gaze during the Posner task may be necessary. Third, participants’ brain function was not evaluated using functional magnetic resonance imaging or other methods; thus, we could not examine the relationship between the reaction time and the activity of the brain regions examined. In this study, we used the results of previous studies as a basis for our discussion. Thus, the brain functions of the participants must be measured. Finally, the MMSE scores of the participants were obtained only up to 2 weeks postoperatively, and whether the decrease in bottom-up attention associated with surgery affects the long-term decline in cognitive function is unclear. Thus, the long-term progress of MMSE in the participants must be evaluated, and the relationship between the decline in bottom-up attention and cognitive function expressed by MMSE should be clarified. We believe that the results of this study will contribute to the development of evaluation and intervention methods for the prevention of cognitive decline in older people.

## Author contributions

**Conceptualization:** Kazuya Akiyama, Shinta Takeuchi, Yusuke Nishida.

**Investigation:** Kazuya Akiyama.

**Methodology:** Kazuya Akiyama, Shinta Takeuchi, Yusuke Nishida.

**Project administration:** Kenichiro Takeshima.

**Supervision:** Kenichiro Takeshima, Yusuke Nishida.

**Writing** – **original draft:** Kazuya Akiyama.

**Writing** – **review & editing:** Kazuya Akiyama, Shinta Takeuchi, Kenichiro Takeshima, Yusuke Nishida.
